# Immune Remodeling Across Advanced Heart Failure, LVAD Support, and Heart Transplantation: From Cellular Activation to Tissue-Resident Memory

**DOI:** 10.3390/cells15211918

**Published:** 2026-10-22

**Authors:** Zbigniew Krasiński, Tomasz Urbanowicz, Mariusz Kowalewski, Giuseppe Maria Raffa, Beata Krasińska, Calogera Pisano, Paolo Manca, Mansur Rahnama, Piotr Suwalski, Krzysztof Gosik, Ewelina Grywalska

**Affiliations:** 1 Department of Vascular, Endovascular Surgery, Angiology and Phlebology, Poznan University of Medical Science, ½ Dluga Street, 61-848 Poznan, Poland; 2 Cardiac Surgery and Transplantology Department, Poznan University of Medical Sciences, ½ Długa, 61-848 Poznań, Poland; 3 Thoracic Research Center, Collegium Medicum, Nicolaus Copernicus University, 85-090 Bydgoszcz, Poland; 4 Department of Precision Medicine in Medical Surgical and Critical Area (Me.Pre.C.C.), University of Palermo, 90134 Palermo, Italy; 5 Department of Cardiac Surgery, Center of Postgraduate Medical Education, Central Clinical Hospital of the Ministry of Interior, 137 Wołowska Street, 02-507 Warszawa, Poland; 6 Department of Research, IRCCS ISMETT, Via Ernesto Tricomi, 5, 90127 Palermo, Italy; 7 Department of Hypertensiology, Angiology, and Internal Medicine, Poznan University of Medical Sciences, ½ Dluga Street, 61-848 Poznań, Poland; 8 Department of Dental Surgery, Medical University of Lublin, 6 Chodźki Street, 20-093 Lublin, Poland; 9 Department of Experimental Immunology, Faculty of Medical Sciences, Medical University of Lublin, 4a Chodźki Street, 20-093 Lublin, Poland

**Keywords:** advanced heart failure, heart transplantation, left ventricular assist device, alloimmunity, immune remodeling, T-cell memory, tissue-resident memory, donor-specific antibodies, single-cell transcriptomics, spatial immunology

## Abstract

**Highlights:**

**What are the main findings?**

• Advanced heart failure, LVAD support, and heart transplantation can be conceptualized as a continuous immunological trajectory rather than isolated clinical stages; we introduce the concept of the “recipient immune biography” to describe how cumulative inflammatory, infectious, device-related, and sensitizing exposures shape the immune state encountered by the donor graft.
• Post-transplant alloimmunity is determined not only by donor-antigen recognition but also by immune-cell fate, clonality, memory, and tissue localization, integrating effector, regulatory, exhaustion-associated, Tfh–B-cell–humoral, and tissue-resident memory programs.

**What are the implications of the main findings?**

• LVAD support may be viewed as an immunological interphase in which heart-failure-associated immune abnormalities may resolve, persist, or be remodeled, potentially modifying the immune substrate subsequently exposed to donor alloantigens.
• Future immune surveillance should move beyond isolated circulating biomarkers toward longitudinal, multimodal phenotyping that integrates blood biomarkers, high-dimensional cellular profiling, single-cell transcriptomics, TCR/BCR clonotyping, graft molecular analysis, and spatial immunology.

**Abstract:**

Advanced heart failure (HF) left ventricular assist device (LVAD) support and heart transplantation (HTx) are commonly considered distinct clinical stages, although each occurs within an immune system shaped by preceding inflammatory and antigenic exposures. This review introduces the concept of the recipient immune biography, describing the cumulative immunological history that accompanies patients from advanced HF through mechanical circulatory support to transplantation. We examine how HF-associated innate and adaptive immune activation may be modified by mechanical unloading while simultaneously being reshaped by blood–device interactions, hemolysis, complement activation, infection, transfusion, and sensitization during LVAD support. Following transplantation, this preconditioned immune repertoire encounters persistent donor alloantigen through direct, indirect, and semidirect allorecognition. We integrate T-cell effector, regulatory, exhaustion-associated, and memory states with T follicular helper–B-cell interactions, plasma-cell persistence, donor-specific antibodies, and tissue-resident immunity. Emphasis is placed on the limitations of peripheral biomarkers for representing graft-localized immune processes. We propose a Longitudinal Immune Trajectory integrating serial peripheral biomarkers with cellular, clonal, molecular, and spatial profiling to reconstruct patient-specific immune remodeling across the HF–LVAD–HTx continuum.

## 1. Advanced Heart Failure as a State of Immune Priming

Advanced HF is accompanied by sustained innate and adaptive immune activation driven by recurrent myocardial injury, hypoperfusion, congestion, and metabolic stress [1,2]. DAMP-mediated activation of TLR and NLRP3 pathways promotes inflammatory cytokine release and monocyte–macrophage activation, while persistent inflammation may alter effector, regulatory, and antigen-experienced lymphocyte populations [3,4,5]. Thus, patients reaching LVAD implantation or HTx carry a preconditioned immune state shaped by HF, infections, transfusions, interventions, and other sensitizing exposures [6,7]. This pre-transplant state should not, however, be interpreted as necessarily donor-specific or directly alloreactive.

We use the term recipient immune biography as an integrative framework describing the cumulative immunological consequences of myocardial injury, systemic inflammation, infection, transfusion, device exposure, sensitizing events, and therapeutic interventions preceding transplantation. It is not synonymous with adaptive immune memory, trained immunity, immune imprinting, or HLA sensitization, but encompasses their potential contributions to the immune state encountered at advanced surgical therapy. Importantly, we do not propose this as a novel immunological mechanism or a prospectively validated clinical construct; rather, it provides a conceptual framework for integrating established biological processes across the HF–LVAD–HTx continuum.

**Figure 1:**
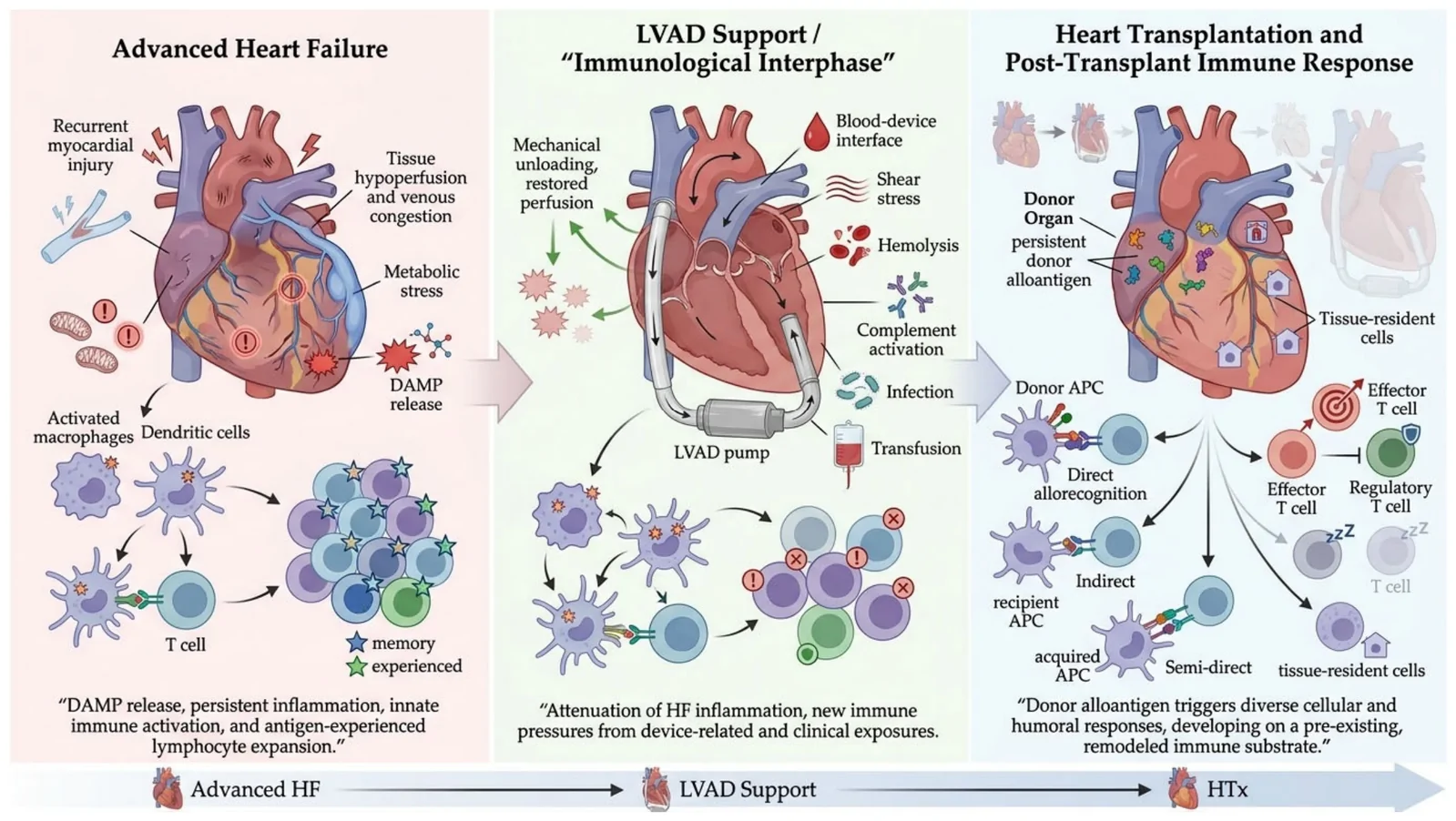
The Recipient Immune Biography: Immune Remodeling Across Advanced Heart Failure, LVAD Support, and Heart Transplantation. The schematic depicts the progressive remodeling of recipient immunity across advanced HF, durable LVAD support, and HTx. In advanced HF, recurrent myocardial injury, metabolic and mitochondrial stress, tissue hypoperfusion, and congestion promote DAMP release and innate immune activation. LVAD support simultaneously removes selected HF-associated inflammatory stimuli and introduces blood–device interactions, altered shear stress, hemolysis, complement activation, infection, and sensitizing exposures. Following HTx, the resulting recipient immune repertoire encounters persistent donor alloantigen through direct, indirect, and semidirect allorecognition, generating competing effector, regulatory, memory, and tissue-resident programs. Symbols indicating cellular activation, dysfunction/apoptosis, and mitochondrial stress are defined directly within the figure. Abbreviations: APC, antigen-presenting cell; DAMP, damage-associated molecular pattern; HF, heart failure; HTx, heart transplantation; LVAD, left ventricular assist device; T cell, T lymphocyte. Created by T Urbanowicz on 25 September 2026 using www.figurelabs.ai (certificate ID: FL-PUB-20260924-6TIQ4K).

This framework is biologically relevant because antigen-experienced lymphocytes differ from naïve cells in activation thresholds, costimulatory dependence, trafficking, and effector capacity [8,9]. LVAD support further reshapes this pre-existing immune state through the opposing effects of hemodynamic recovery and new device-related stimuli, including biomaterial exposure, altered shear, hemolysis, complement activation, and infection. HTx subsequently introduces persistent donor alloantigen into this already conditioned immune environment [6,10]. Whether components of the preceding phenotype persist sufficiently to influence subsequent alloimmunity remains unresolved (Figure 1).

This narrative review was informed by a structured search of PubMed/MEDLINE, Scopus, and Web of Science for English-language publications through September 2026 that address immune remodeling in advanced HF, durable LVAD support, and HTx. Cardiac-specific human evidence was prioritized and supplemented by mechanistically informative non-cardiac transplantation and experimental studies when corresponding cardiac data were limited. Reference lists of relevant primary studies and reviews were additionally screened. Study selection was narrative and emphasized biological relevance, clinical applicability, and mechanistic contribution; no formal meta-analysis, risk-of-bias assessment, or quantitative evidence grading was performed.

## 2. LVAD as a Driver of Immune Remodeling

Left ventricular assist device (LVAD) implantation represents a major transition in the immune trajectory of advanced heart failure (HF) [11]. Mechanical unloading restores cardiac output, improves end-organ perfusion, reduces venous congestion, and partially removes several stimuli responsible for chronic immune activation in the failing circulation [12]. At the same time, however, durable support introduces a permanent blood-contacting artificial surface, non-physiological shear forces, hemolysis, altered hemostatic signaling, complement activation, and a persistent risk of device-related infection [13,14,15]. The resulting phenotype is likely to reflect the balance between these opposing forces and may evolve substantially with duration of support.

Mechanical unloading attenuates upstream inflammatory drivers, including hypoxia, congestion, neurohormonal activation, and recurrent myocardial stress, and longitudinal HM3 studies have documented reductions in CRP and selected inflammatory cytokines [12]. Conversely, persistent blood–device contact activates platelet, leukocyte, coagulation, and complement pathways, while non-physiological shear promotes cellular injury [13,16].

Protein adsorption, complement-derived C3a/C5a, and DAMP release—including ATP, mitochondrial DNA, and HMGB1—may engage TLR–MYD88–NF-κB and NLRP3 signaling, linking hemocompatibility-related injury to innate inflammation. LVAD support should therefore be viewed neither as uniformly pro- nor anti-inflammatory, but as a dynamic balance between resolution of HF-associated inflammatory signaling and device-conditioned immune stimulation.

Historical LVAD studies have described monocyte and T-cell activation, increased CD95/Fas expression, activation-induced CD4+ T-cell death, impaired proliferative responses, and susceptibility to opportunistic infection [17,18]. Subsequent continuous-flow studies demonstrated broader changes across innate and adaptive compartments, although contemporary observations indicate substantial interindividual and temporal heterogeneity [19,20]. Importantly, contemporary observations suggest that these immune phenotypes are heterogeneous and may evolve with duration of support, while the pre-implant immune state itself appears to influence subsequent susceptibility to infection [21].

Contemporary durable support is dominated by the fully magnetically levitated HeartMate 3 (HM3), whose flow characteristics and reduced hemolytic burden differ substantially from earlier pulsatile and axial-flow systems [22,23]. Consequently, the marked CD95-mediated lymphocyte apoptosis and immune dysfunction reported in historical cohorts cannot automatically be extrapolated to contemporary support. Interpretation across device generations is further complicated by differences in support duration and cumulative exposure to infection, bleeding, transfusion, and other sensitizing events.

The principal immune transitions across advanced HF, historical and contemporary LVAD support, and subsequent heart transplantation are summarized in Table 1.

Device-related infection adds sustained microbial stimulation to the LVAD immune environment and may reinforce innate activation while altering adaptive differentiation and memory [36,37].

More broadly, LVAD-associated immune remodeling appears heterogeneous: inflammatory activation may coexist with regulatory adaptation, impaired cellular responsiveness, or partial recovery as HF-related inflammatory stimuli recede [19,20]. It remains unknown whether these changes persist at the transcriptional, epigenetic, or clonal level after prolonged support. This distinction is important: transient adaptation to LVAD support should not be equated with immune reprogramming.

**Figure 2:**
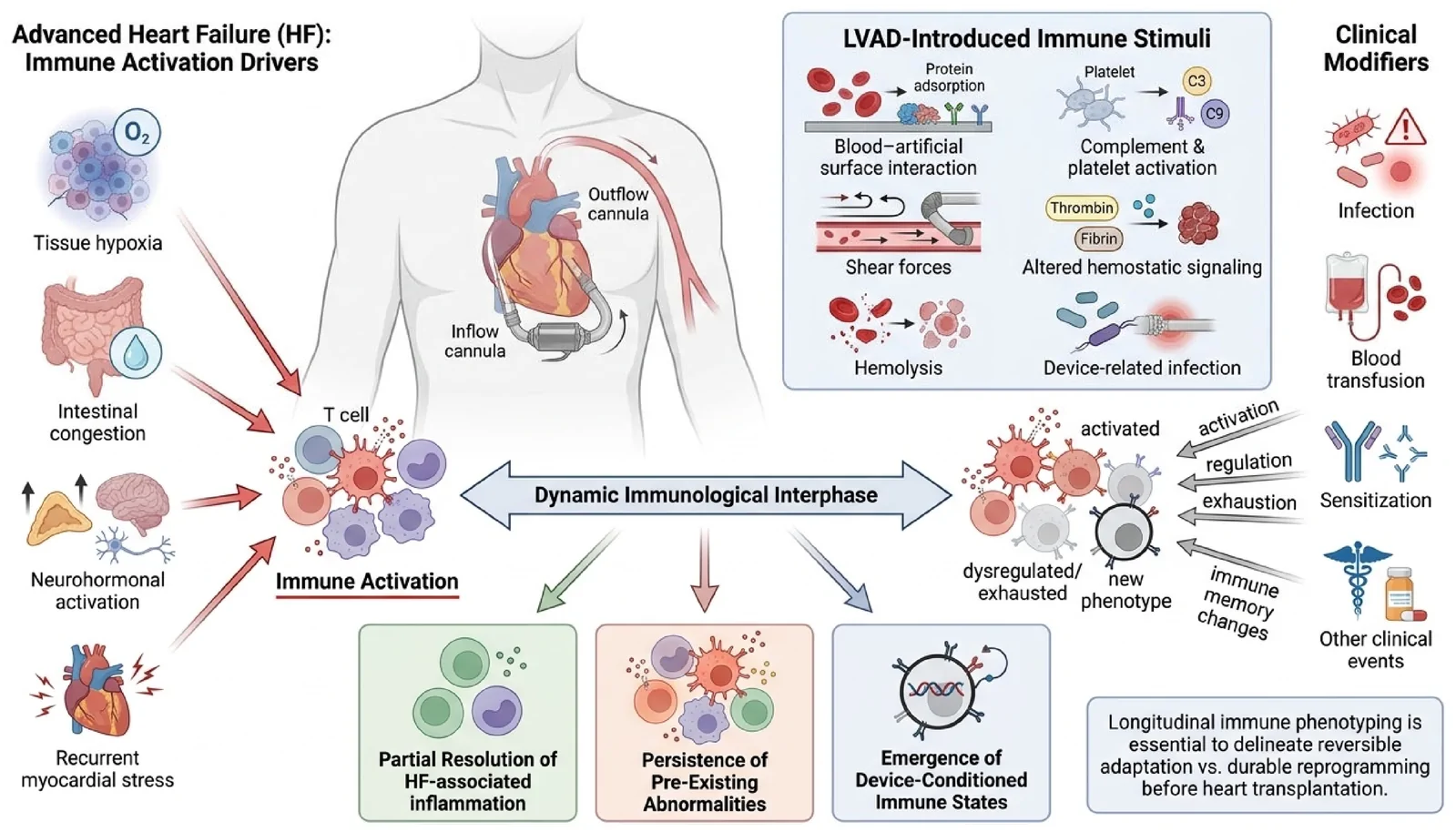
LVAD Support as an Immunological Interphase: Resolution, Persistence, and Remodeling of the Pre-Existing Immune State. The schematic conceptualizes LVAD support as a dynamic immunological interphase rather than a uniformly pro- or anti-inflammatory state. Mechanical unloading and restoration of systemic perfusion may attenuate selected HF-associated inflammatory drivers, whereas persistent blood–device contact, altered shear forces, hemolysis, complement and platelet activation, infection, transfusion, and sensitizing events introduce new immune stimuli. Their interaction may result in partial resolution of HF-associated inflammation, persistence of pre-existing abnormalities, or emergence of device-conditioned immune states. These trajectories are not mutually exclusive and may evolve with duration of support and intervening clinical events. Abbreviations: C3, complement component 3; C9, complement component 9; HF, heart failure; LVAD, left ventricular assist device; O_2_, oxygen; T cell, T lymphocyte. Created by T Urbanowicz on 24 September 2026 using www.figurelabs.ai (certificate ID: FL-PUB-20260924-6TIQ4K).

Resolving this question will require longitudinal phenotyping from pre-implant HF through stable LVAD support and, where applicable, transplantation [38,39] (Figure 2).

## 3. The Cellular Transition from Immune Priming to Alloreactivity

Heart transplantation introduces a qualitatively different immunological challenge into an immune system already conditioned by advanced heart failure and, in many recipients, by prolonged mechanical circulatory support [40]. Unlike the sterile inflammatory signals that dominate during progressive myocardial injury, transplantation provides a persistent source of highly immunogenic non-self HLA molecules, together with tissue injury resulting from donor management, organ preservation, ischemia–reperfusion, and surgical implantation [41,42]. Transplantation marks the point at which this preceding immune history is confronted with donor specificity [43].

At the cellular level, productive T-cell activation requires the integration of three interconnected signals. Recognition of peptide–HLA complexes by the T-cell receptor (TCR) provides antigen specificity, but TCR engagement alone is insufficient to determine the magnitude or functional character of the ensuing response. A second, costimulatory signal is classically provided through CD28 engagement by CD80/CD86 expressed on antigen-presenting cells (APCs), allowing antigen recognition to progress toward effective activation rather than anergy or incomplete responsiveness. A third layer is provided by the surrounding cytokine milieu, which shapes subsequent differentiation, expansion, and functional polarization of the responding T-cell population [44]. TCR engagement, costimulation, and cytokine-dependent signaling converge on intracellular programs governing clonal expansion, metabolic adaptation, survival, and differentiation. The integrated strength, duration, and context of these signals influence whether donor-reactive clones preferentially acquire effector, regulatory, memory, or dysfunction/exhaustion-associated states [45,46]. Allorecognition determines how donor antigen is encountered, whereas intracellular signal integration helps determine the subsequent functional fate of the responding clone.

Donor alloantigens are encountered through direct, indirect, and semidirect allorecognition. Direct recognition of intact donor HLA–peptide complexes contributes prominently to early alloimmunity, whereas indirect presentation of processed donor antigens by recipient APCs may sustain CD4+ T-cell help, humoral immunity, and chronic graft injury. Semidirect presentation, in which recipient APCs acquire intact donor HLA, provides an additional bridge between these pathways [41,42,47,48]. Classical sensitizing events—including transfusion, pregnancy, prior transplantation, and mechanical circulatory support—add further heterogeneity [49]. Thus, donor alloantigen encounters a recipient repertoire shaped by prior cellular memory, clonality, and humoral sensitization rather than an immunologically neutral host.

## 4. Cell Fate Matters: Effector Function, Regulation, Exhaustion, and Memory

Recognition of donor antigen initiates alloimmunity but does not, by itself, determine its biological consequence. Following activation, alloreactive T cells enter differentiation programs shaped by the strength and persistence of TCR signaling, costimulation, cytokine exposure, metabolic conditions, and tissue-derived signals [50,51].

These influences determine whether responding cells predominantly acquire effector, regulatory, dysfunction/exhaustion-associated, or memory properties. This distinction is particularly relevant after transplantation because, unlike a self-limited infection, the principal source of alloantigen persists for the lifetime of a functioning graft. Immune control must therefore be maintained in the continued presence of the donor organ [52,53].

### 4.1. Effector T-Cell Programs

Productive alloimmune activation generates complementary CD4+ and CD8+ effector programs. Th1- and Th17-associated responses amplify graft inflammation through IFN-γ- dependent macrophage activation, enhanced antigen presentation, chemokine production, and leukocyte recruitment, whereas activated CD8+ T cells exert direct cytotoxicity via perforin–granzyme and death receptor pathways. Single-cell studies of cardiac allograft rejection confirm the expansion of proliferative and cytotoxic CD8+ populations, accompanied by IFN-γ-, TNF-, granzyme-, and chemokine-associated programs [54]. Within the graft, these responses converge on shared cellular targets and interact closely with macrophages, endothelial cells, fibroblasts, and other tissue-resident populations [55,56].

### 4.2. Regulatory T Cells and Counter-Regulatory Control

Counter-regulatory mechanisms operate simultaneously. Regulatory T cells (Tregs), particularly CD4+CD25^high^FOXP3+ populations, restrain conventional T-cell responses through complementary mechanisms involving CTLA-4-dependent modulation of costimulation, IL-10 and TGF-β signaling, and CD39–CD73-mediated adenosine generation [57,58]. Peripheral Treg abundance alone does not establish suppressive competence, stability, tissue localization, or the functional balance between regulatory and effector populations [59,60].

### 4.3. Inhibitory Signaling and Exhaustion-Associated T-Cell States

Persistent TCR engagement may progressively induce inhibitory receptors, transcriptional and metabolic remodeling, and reduced proliferative or cytokine-producing capacity [61,62]. PD-1, TIM-3, LAG-3, and TIGIT are associated with such states, but none independently establishes exhaustion; PD-1, in particular, may reflect recent activation rather than irreversible dysfunction [63,64]. In the setting of persistent alloantigen, inhibitory signaling may restrain effector activity and contribute to the equilibrium required for long-term graft survival, as indirectly illustrated by the rejection risk associated with immune checkpoint inhibition in solid-organ recipients [65,66,67].

The term “exhaustion” should therefore be used cautiously after transplantation. Reduced function or inhibitory receptor expression may reflect progressive dysfunction, adaptive restraint, pharmacological immunosuppression, or overlapping states [65,68,69]. Defining transplantation-associated exhaustion consequently requires functional and molecular evidence rather than inference from surface phenotype alone [61].

The principal T-cell states and their interpretative limitations are summarized in Table 2. These states should be regarded as dynamic and potentially overlapping rather than fixed identities: persistent alloantigen may drive individual clones through effector, regulatory, inhibitory, and memory-associated programs over time.

### 4.4. Circulating and Tissue-Resident Memory T Cells

Central-memory T cells retain substantial proliferative reserve, whereas effector-memory populations support rapid peripheral responses [53,86]. Tissue-resident memory T cells (T_RM), by contrast, persist within non-lymphoid tissues and can respond locally to antigen without repeated recruitment from blood [52,53,87]. Peripheral phenotyping may therefore underestimate the organization and persistence of donor-reactive memory within the graft [62,88].

Post-transplant memory develops on a pre-existing antigen-experienced repertoire shaped by previous inflammatory and sensitizing exposures. Heterologous immunity may permit pre-existing clones to cross-react with allogeneic HLA, while subsequent donor-antigen exposure can select and expand donor-reactive clonotypes [12,89,90]. Clonality and functional state should be considered complementary rather than interchangeable dimensions of alloimmune memory.

**Figure 3:**
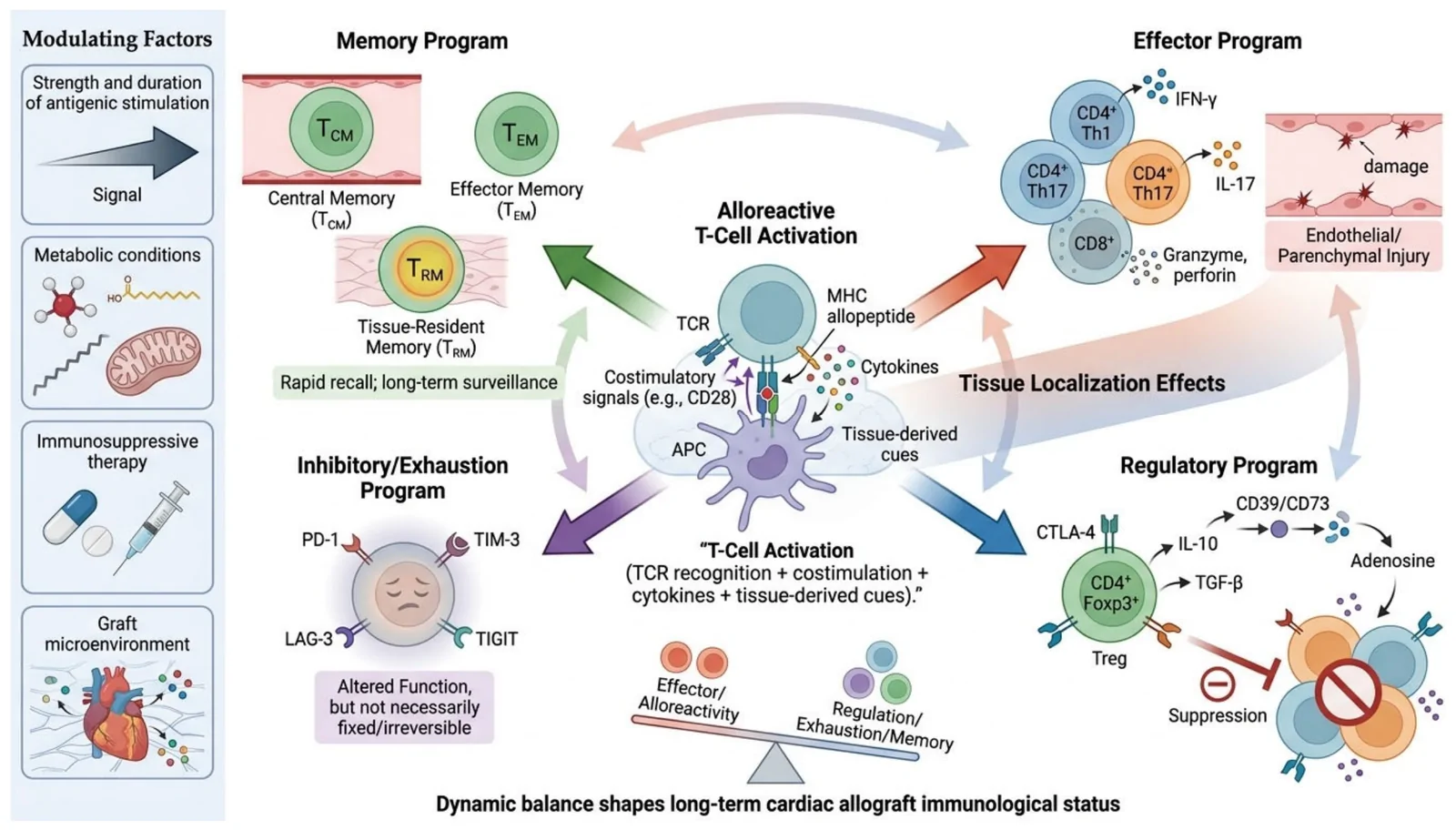
Cell Fate After Alloantigen Recognition: From Effector Activation to Regulation, Exhaustion, and Memory. The schematic illustrates how alloantigen recognition translates into divergent yet potentially overlapping T-cell fates. Integration of TCR signaling, costimulation, cytokine exposure, metabolic conditions, immunosuppressive therapy, and graft-derived cues may promote effector, regulatory, inhibitory/exhaustion-associated, or memory programs. These states should not be interpreted as fixed or mutually exclusive phenotypes; persistent donor-antigen exposure may permit dynamic transitions among them over time. Tissue localization further modifies their biological consequences, linking cellular state to endothelial and parenchymal injury, immune regulation, or durable immune surveillance within the graft. Abbreviations: APC, antigen-presenting cell; CD, cluster of differentiation; CTLA-4, cytotoxic T-lymphocyte-associated protein 4; IFN-γ, interferon gamma; IL, interleukin; LAG-3, lymphocyte-activation gene 3; MHC, major histocompatibility complex; PD-1, programmed cell death protein 1; T_CM, central memory T cell; TCR, T-cell receptor; T_EM, effector memory T cell; TGF-β, transforming growth factor beta; Th1, T helper 1 cell; Th17, T helper 17 cell; TIGIT, T-cell immunoreceptor with immunoglobulin and ITIM domains; TIM-3, T-cell immunoglobulin and mucin-domain containing-3; Treg, regulatory T cell; T_RM, tissue-resident memory T cell. Created by T Urbanowicz on 24 September 2026 using www.figurelabs.ai (certificate ID: FL-PUB-20260924-18CC9Q).

Critically, clonality and functional state are not interchangeable. Related alloreactive cells may occupy different transcriptional, metabolic, or functional states according to antigen exposure, regulatory pressure, and tissue localization. Effector, regulatory, dysfunction/exhaustion-associated, and memory programs may therefore coexist within the same recipient—and potentially within the same graft—as evolving cellular trajectories rather than mutually exclusive terminal phenotypes (Figure 3).

## 5. Adaptive Immune Memory Beyond T Cells: Tfh–B-Cell–Plasma-Cell–DSA Axis

### 5.1. Tfh–B-Cell Cooperation and Germinal-Center Responses

Adaptive immune memory after HTx extends beyond antigen-experienced T cells to the Tfh–B-cell axis. Tfh-derived CD40L and cytokine signals, particularly IL-21, support donor-reactive B-cell activation, germinal-center selection, affinity maturation, class switching, and differentiation into memory B cells and antibody-secreting populations [91,92,93,94]. Because donor alloantigen persists, these processes may remain active despite immunosuppression, with the balance between persistence and quiescence shaped by antigen exposure and regulatory mechanisms.

Within germinal centers, somatic hypermutation, affinity-dependent selection, and class-switch recombination progressively reshape the responding repertoire [72,95]. Because the allograft provides a persistent alloantigen, this process occurs in an environment of continuing antigenic exposure in which T-cell help, immunosuppression, and regulatory mechanisms jointly determine whether B-cell clones persist, mature, become quiescent, or undergo further differentiation [96].

### 5.2. Memory B Cells and Long-Lived Plasma Cells

Memory B cells and long-lived plasma cells represent biologically distinct forms of humoral persistence. Memory B cells preserve donor-reactive recall capacity and can rapidly generate antibody-secreting progeny after renewed stimulation, whereas long-lived plasma cells may maintain antibody production with limited dependence on renewed B-cell activation [97,98]. Consequently, serum antibody measurements cannot reconstruct the size, clonal diversity, or recall potential of the underlying memory B-cell compartment [99,100]. Conversely, depletion of circulating B cells may leave established long-lived plasma-cell populations relatively unaffected [101].

### 5.3. DSA and Antibody-Mediated Effector Mechanisms

Preformed DSA reflects sensitization preceding transplantation, whereas de novo DSA (dnDSA) indicates the emergence or renewed detectability of a donor-directed humoral response after transplantation [102,103]. Persistent class II DSA, particularly HLA-DQ antibodies, has been associated with adverse long-term cardiac allograft outcomes [104]. Yet DSA detection alone incompletely defines pathogenic potential, which is influenced by specificity, persistence, subclass, avidity, complement-binding capacity, and antigen density and accessibility within the graft [105]. Mean fluorescence intensity should likewise be interpreted with caution, as it is a semiquantitative assay signal rather than a direct measure of antibody concentration or pathogenicity [106,107].

DSA connects adaptive humoral memory to tissue-level effector mechanisms through the graft endothelium. Binding to donor HLA can activate complement and promote endothelial activation, altered vascular permeability, leukocyte recruitment, and microvascular inflammation [108,109].

Antibody-coated vascular targets may simultaneously engage FcγRIIIa/CD16a on NK cells, thereby coupling donor specificity to antibody-dependent cellular cytotoxicity and inflammatory signaling [110,111]. Complement activation, NK-cell responses, endothelial signaling, and myeloid recruitment may therefore operate concurrently rather than as isolated mechanisms [112]. Consistent with this integrated model, molecular studies of antibody-mediated rejection identify prominent NK-cell- and IFN-γ-associated signatures, including in some cases without detectable circulating DSA [113].

The cellular architecture linking donor-antigen recognition to humoral memory and graft injury is summarized in Table 3. The central implication is that circulating DSA represents a downstream product of a broader cellular network rather than a complete representation of the donor-specific humoral state.

Repeated endothelial activation provides a plausible continuum between donor-specific humoral memory, microvascular inflammation, antibody-mediated rejection, and chronic vascular remodeling [126,127,128]. These processes may remain biologically active despite preserved graft function, emphasizing that clinical stability does not necessarily imply immunological quiescence.

Accordingly, integrating antibody phenotypes with BCR/TCR clonality and tissue-resolved cellular profiling may better reconstruct the cellular origins and persistence of humoral alloimmunity than DSA measurement alone [129,130].

Throughout the following sections, evidence derived directly from human heart transplantation is distinguished from mechanistic observations obtained in experimental models or other solid-organ transplants; the latter are used to define biologically plausible mechanisms rather than to imply cardiac-specific validation.

## 6. The Transplanted Heart as a Tissue Immune Ecosystem

Following transplantation, the heart becomes a dynamic immune ecosystem in which donor-derived cells interact with infiltrating recipient leukocytes, endothelium, fibroblasts, and other stromal populations [131,132]. These interactions shape antigen recognition, immune regulation, cytotoxicity, antibody-mediated injury, repair, and vascular remodeling [53,133]. The graft should therefore be viewed not merely as a target of systemic alloimmunity, but as a spatially organized immune–vascular–stromal compartment whose biology depends on cellular identity, origin, state, and localization [134].

In contrast to circulating memory populations, tissue-resident memory (T-RM) cells exemplify this principle by remaining positioned within the graft. T-RM may persist within the graft and provide a locally positioned memory compartment capable of rapid antigen re-encounter [135]. Their contribution to human cardiac allograft biology remains incompletely defined.

### 6.1. Macrophages and the Myeloid Compartment

The graft macrophage compartment evolves dynamically from donor-resident populations toward increasing recipient monocyte-derived chimerism. Experimental cardiac transplantation suggests functional divergence between donor CCR2− and CCR2+ macrophages, with the latter favoring inflammatory recruitment and the former potentially supporting graft homeostasis [136]. Human single-nucleus studies similarly indicate progressive replacement of donor-resident macrophages by recipient-derived populations capable of inflammatory, reparative, or profibrotic states [137]. This temporal and functional heterogeneity renders simple M1/M2 classification inadequate [138]. Macrophages integrate injury sensing, antigen presentation, interferon and complement signaling, phagocytosis, inflammation, and repair [139]. Their biological significance depends less on abundance than on origin, transcriptional state, localization, and participation in multicellular graft networks [140,141].

### 6.2. NK Cells and Antibody-Dependent Effector Responses

Natural killer (NK) cells occupy a strategically important interface between humoral recognition and cellular tissue injury. Engagement of FcγRIIIa (CD16a) by IgG bound to donor vascular targets can induce NK-cell degranulation, cytokine release, and antibody-dependent cellular cytotoxicity, thereby providing a cellular effector pathway downstream of donor-specific antibodies [142]. This mechanism is particularly relevant within the microvasculature, where NK-cell activation is closely associated with antibody-mediated rejection and microvascular inflammation [124,143]. NK-derived IFN-γ may further amplify endothelial and myeloid activation, extending the consequences of antibody recognition beyond the initial Fc receptor interaction [144]. Notably, molecular studies identify NK-cell-associated and IFN-γ-associated programs in antibody-mediated rejection even when circulating DSA are not detected [145]. NK cells should be viewed within a broader vascular immune circuit that integrates antibody binding, endothelial activation, Fc receptor signaling, and local myeloid responses; their tissue activation and localization may be more informative than their peripheral blood abundance.

### 6.3. Fibroblasts and Vascular Smooth Muscle Cells

The stromal and vascular compartments are equally active participants in this ecosystem. Fibroblasts respond to inflammatory and tissue-injury signals by remodeling extracellular matrix and producing cytokines, chemokines, and growth factors that influence immune-cell recruitment and persistence [53]. Signals derived from T cells and macrophages promote fibroblast activation, creating reciprocal circuits in which inflammation and repair become progressively intertwined [146]. Within the vascular wall, inflammatory signaling can shift VSMCs from a contractile phenotype toward proliferative, migratory, and matrix-remodeling states that directly contribute to neointimal expansion in Cardiac allograft vasculopathy (CAV) [147].

### 6.4. Immune–Stromal Cellular Neighborhoods and CAV

Single-cell and spatial studies increasingly resolve these processes as multicellular tissue neighborhoods rather than independent cellular compartments. Macrophages, activated fibroblasts, endothelial cells, pericytes, lymphocytes, and phenotypically altered vascular cells may occupy spatially organized niches in which inflammatory and remodeling programs coexist, particularly around the graft microvasculature [116,120]. Chronic graft injury is therefore unlikely to follow a simple linear sequence in which inflammation ends before fibrosis begins. Instead, persistent immune activation, endothelial injury, stromal activation, and vascular remodeling may evolve in parallel and reinforce one another through reciprocal cell–cell signaling [148]. Such networks provide a plausible biological mechanism by which repeated or incompletely resolved alloimmune injury is translated into progressive fibrosis and CAV [118].

The principal immune, vascular, and stromal populations that constitute the transplanted-heart ecosystem are summarized in Table 4. Their biological relevance resides not simply in cellular identity or abundance, but in the convergence of origin, functional state, spatial localization, and cellular interaction within myocardial and vascular niches.

**Figure 4:**
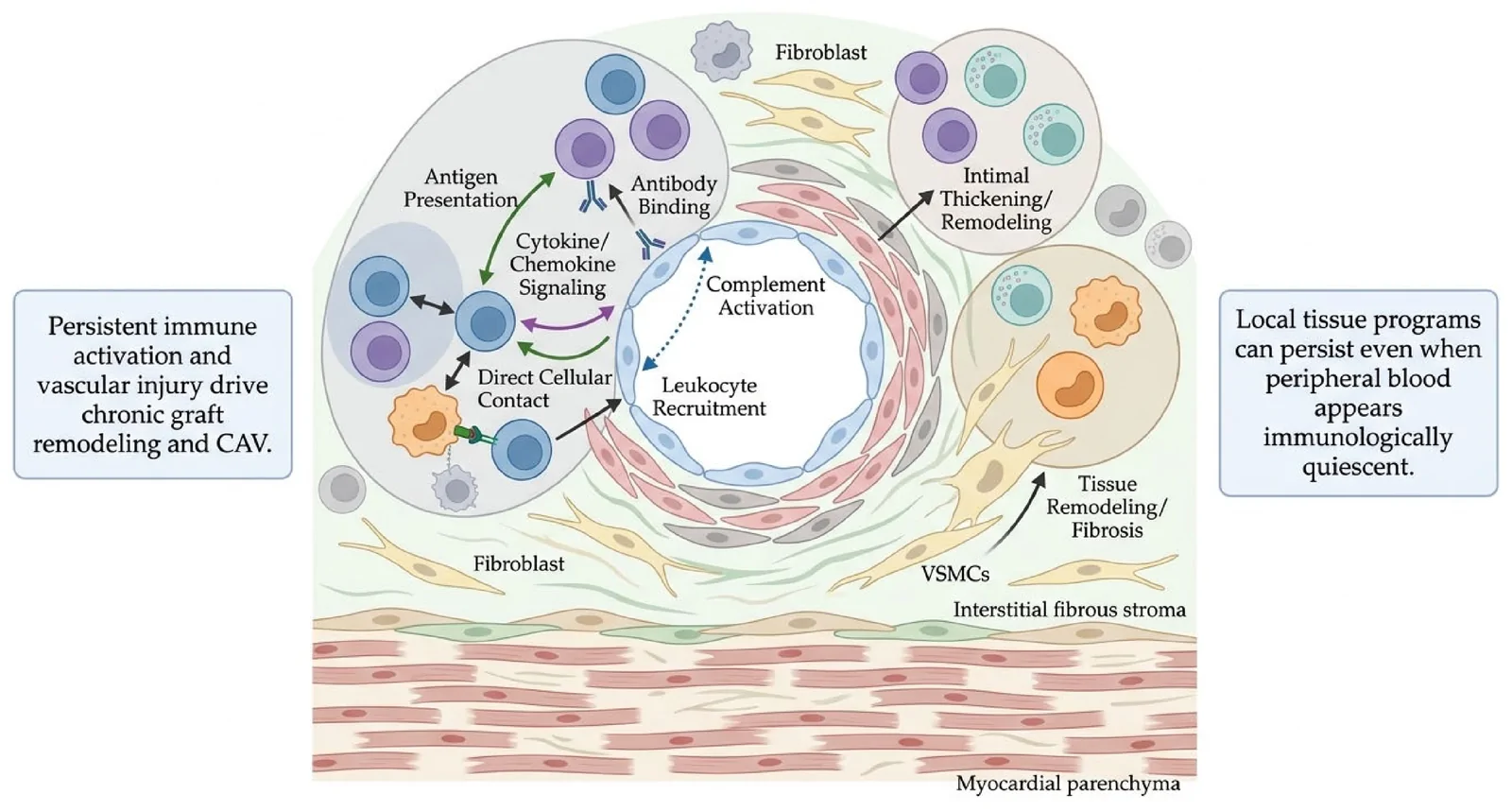
Transplanted Heart as an Immune–Vascular–Stromal Ecosystem. The schematic depicts the cardiac allograft as an integrated immune–vascular–stromal ecosystem in which alloimmune activation, endothelial injury, complement activity, leukocyte recruitment, and stromal remodeling are interconnected rather than sequential processes. T cells, antibody-mediated mechanisms, and innate immune populations interact with endothelial cells, fibroblasts, and VSMCs to generate multicellular tissue niches that sustain inflammation, extracellular matrix remodeling, intimal expansion, and fibrosis. Such local programs may persist despite apparent peripheral or histological quiescence and provide a biological framework linking recurrent immune injury to progressive CAV and chronic graft remodeling. Abbreviations: CAV, cardiac allograft vasculopathy; VSMCs, vascular smooth muscle cells. Created by T Urbanowicz on 24 September 2026 using www.figurelabs.ai (certificate ID: FL-PUB-20260924-BI9CYI).

The characteristic concentric intimal expansion of donor coronary arteries can be viewed as the anatomical consequence of this prolonged multicellular dialogue. Adaptive and innate immune responses activate the endothelium and promote leukocyte recruitment; T cells, NK cells, and macrophages provide cytotoxic and cytokine signals; fibroblasts and vascular cells alter their phenotype, proliferative behavior, and extracellular matrix production; and the remodeled tissue environment subsequently modifies immune cell recruitment and function. As illustrated in Figure 4, alloimmune activation, endothelial injury, innate effector responses, and stromal remodeling are therefore better understood as an interconnected network that progressively reorganizes the graft microenvironment rather than as sequential, independent events.

Peripheral profiling nevertheless captures only part of this biology. Single-cell RNA sequencing of peripheral blood from 40 heart-transplant recipients identified 745 cell-specific differentially expressed genes in high-grade CAV, along with modest expansion of CD4+ central memory T cells and CD14+ and CD16+ monocytes [154]. These observations demonstrate a systemic immune signature of CAV but cannot reconstruct the coronary lesion itself: circulating cells provide a correlate of tissue disease rather than its spatial architecture. Spatial transcriptomic studies of human cardiac biopsies further reveal molecular heterogeneity within conventional rejection categories involving T cells, endothelial cells, fibroblasts, and myeloid populations [39]. Histologically similar biopsies may therefore represent biologically distinct molecular states and potentially different trajectories.

This distinction shifts the relevant unit of biological interpretation from the individual cell toward the multicellular neighborhood. A macrophage-derived chemokine acquires greater mechanistic meaning when its receptor-bearing lymphocyte occupies the same vascular niche; analogous relationships apply to growth-factor signaling between immune cells, fibroblasts, and VSMCs [155]. Immune populations shape endothelial and stromal behavior, while resident graft cells, in turn, regulate leukocyte recruitment, retention, and differentiation [156]. Once established, such networks may sustain fibrosis and vascular remodeling even after overt rejection has subsided [123], potentially explaining why established structural programs cannot necessarily be reversed by intensifying conventional lymphocyte-directed immunosuppression.

This tissue-centered perspective also changes the interpretation of apparent clinical stability. A biopsy without active cellular rejection may still contain endothelial, macrophage, fibroblast, and VSMC programs established by previous injury. Histology captures the dominant pattern at a particular moment; late graft disease reflects the accumulated consequences of immune injury, adaptation, and incomplete repair. Accordingly, preserved graft function and absence of overt rejection should not be equated with tissue-level immunological quiescence.

## 7. From Peripheral Biomarkers to Spatial Immunology: Toward Precision Immune Phenotyping

Contemporary immune profiling can interrogate circulating biomarkers, cellular phenotypes, alloantibodies, clonal repertoires, transcriptional states, and spatially resolved graft populations at unprecedented resolution [62]. The principal challenge is therefore no longer the generation of additional biomarkers, but their biological integration across compartments and over time to reconstruct the evolving recipient–graft immune state [157].

### 7.1. Conventional and Emerging Circulating Biomarkers

Endomyocardial biopsy, DSA, and dd-cfDNA interrogate related, but distinct dimensions of graft biology: histological injury, donor-directed humoral recognition, and graft-cell injury, respectively. None alone establishes the complete immune state. Precision phenotyping should therefore determine which biological process an abnormality represents, where it occurs, and how it evolves relative to the recipient’s preceding immune state.

Beyond DSA and dd-cfDNA, several complementary biomarker classes may provide information on biological processes that are incompletely captured by conventional rejection surveillance. Non-HLA antibodies and autoantibodies directed against vascular, endothelial, or intracellular antigens have been associated with graft injury in selected transplant populations and may identify humoral activity not explained by conventional anti-HLA DSA. Their interpretation remains challenging, however, because assay standardization, pathogenic thresholds, antigen specificity, and incremental prognostic value remain heterogeneous. Accordingly, these antibodies should currently be regarded as complementary biological signals rather than interchangeable surrogates for established DSA testing.

Soluble inflammatory mediators provide a different layer of information. Cytokines and chemokines reflecting IFN-γ-, TNF-, IL-6-, IL-1-, and related inflammatory programs may indicate ongoing immune activation but generally lack sufficient graft specificity when interpreted in isolation, particularly in recipients with concurrent infection, systemic inflammation, or tissue injury. Soluble suppression of tumorigenicity 2 (sST2), reflecting myocardial stress and inflammatory–fibrotic signaling, similarly offers potentially useful prognostic information but cannot by itself distinguish alloimmune injury from broader cardiovascular stress. Their principal value may therefore lie in multimarker trajectories rather than isolated thresholds.

Extracellular vesicles (EVs), including exosomes, represent a potentially richer source of graft- and immune-derived molecular information because they transport proteins, lipids, messenger RNA, microRNA, and other nucleic-acid cargo between cells. Donor- or graft-associated EV signatures could theoretically provide information regarding endothelial activation, immune-cell communication, and tissue injury before these processes become apparent through conventional functional measures. Translation remains limited by pre-analytical variability, isolation methodology, vesicle heterogeneity, normalization, and the absence of broadly validated clinical thresholds.

A conceptually distinct biomarker is Torque Teno Virus (TTV) load. Rather than directly measuring graft injury or donor-specific immunity, TTV replication has been investigated as an indirect indicator of the net state of immunosuppression. Higher viral loads may reflect greater pharmacological immune suppression, whereas lower levels may identify relatively preserved immune competence. This distinction is clinically important because immune surveillance after HTx involves two complementary questions: whether graft-directed immune injury is occurring and whether the recipient is globally over- or under-immunosuppressed. Combining injury-associated biomarkers such as dd-cfDNA and DSA with markers reflecting systemic immune competence may therefore provide a more biologically informative framework for individualized immunosuppression than escalation or reduction based on any single assay. Prospective validation remains necessary before such multimarker strategies can be used as stand-alone treatment algorithms.

### 7.2. Peripheral Cellular Phenotyping: Opportunities and Biological Limitations

Peripheral blood provides the practical backbone for such longitudinal assessment because it permits repeated sampling without graft injury [124]. Flow cytometry can resolve activated, regulatory, effector, memory, B-cell, NK-cell, and myeloid populations; soluble mediators reflect systemic inflammatory activity; DSA introduces donor specificity; and gene-expression profiling and dd-cfDNA provide complementary information on immune activation and graft injury. Importantly, these signals are not interchangeable. Clinical studies combining peripheral gene-expression profiling with dd-cfDNA already support the principle that multimodal assessment can distinguish biological states that remain unresolved when either measurement is interpreted alone [158].

The principal limitation of peripheral biomarkers is that they compress complex tissue biology into a circulating signal. Increased dd-cfDNA indicates graft-cell injury without identifying its cellular mechanism [125,145]. DSA establishes donor-directed antibody recognition but cannot determine whether antibody production reflects an active germinal-center response, reactivated memory B cells, or persistent long-lived plasma cells [159]. Likewise, PD-1 expression may accompany activation, adaptive restraint, or progressive dysfunction, while enumeration of regulatory or memory populations provides little information regarding clonality, transcriptional state, or tissue localization. Increasing the number of circulating biomarkers may therefore improve detection without proportionately improving biological interpretation [160].

### 7.3. Single-Cell and Clonal Resolution of the Immune Response

Single-cell RNA sequencing resolves transcriptionally distinct states across lymphoid and myeloid compartments, identifying activation, regulatory, cytotoxic, metabolic, interferon-responsive, and exhaustion-associated programs. Its principal value lies in assigning biological signals to specific cell populations and functional states.

Integration with TCR and BCR sequencing extends this resolution across time by tracing clonal persistence and evolution. T-cell clonotypes may be followed across transplantation-related transitions and into circulating or tissue-resident memory compartments [88,161], while BCR sequencing can distinguish transient plasmablast responses from durable donor-reactive B-cell lineages [162]. These approaches shift immune profiling from static phenotype toward clonal history and cellular fate.

### 7.4. Spatial Immunology and Multilevel Precision Phenotyping

Spatial approaches add a dimension unavailable in blood biomarkers or dissociated single-cell analyses: tissue context. By localizing molecularly defined immune states relative to endothelium, donor antigen, fibroblasts, and vascular smooth muscle cells, they extend interpretation from biomarkers and individual cells to functionally organized multicellular niches.

These analytical levels are complementary rather than competing. As summarized in Table 5, their integration connects circulating signals with cellular phenotype, clonal history, graft-specific molecular programs, and spatially organized tissue interactions.

### 7.5. The Longitudinal Immune Trajectory: Toward Patient-Specific Immune Reconstruction

**Figure 5:**
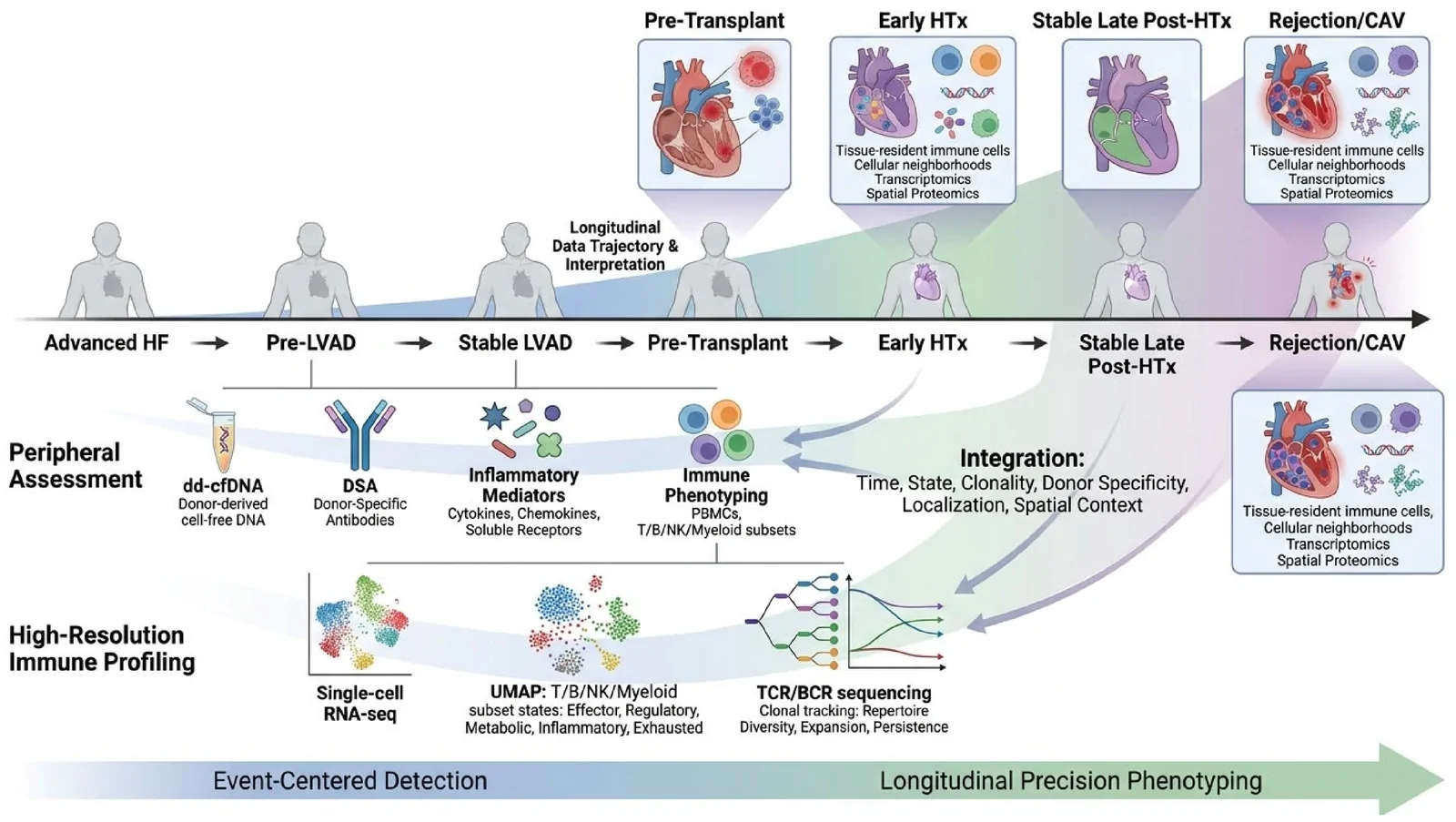
The Longitudinal Immune Trajectory: From Peripheral Biomarkers to Spatial Precision Immunology. The schematic presents the proposed Longitudinal Immune Trajectory as a framework for reconstructing recipient immune remodeling across advanced HF, LVAD support, transplantation, stable post-transplant follow-up, rejection, and CAV. Serial peripheral measurements provide a longitudinal backbone, while selected clinical transitions may be interrogated at greater resolution using single-cell profiling, TCR/BCR clonotyping, graft molecular analysis, and spatial approaches. Integration across time, cellular state, clonality, donor specificity, and tissue localization is intended to distinguish transient immune activation from persistent or progressively remodeled states. The framework is conceptual and hypothesis-generating and should not be interpreted as a validated clinical surveillance algorithm. Abbreviations: BCR, B-cell receptor; CAV, cardiac allograft vasculopathy; dd-cfDNA, donor-derived cell-free DNA; DSA, donor-specific antibody; HF, heart failure; HTx, heart transplantation; LVAD, left ventricular assist device; NK, natural killer; PBMCs, peripheral blood mononuclear cells; RNA-seq, RNA sequencing; TCR, T-cell receptor; UMAP, Uniform Manifold Approximation and Projection. Created by T Urbanowicz on 24 September 2026 using www.figurelabs.ai (certificate ID: FL-PUB-20260924-MJLFPX).

The broader opportunity is to organize these measurements longitudinally rather than episodically. We propose a Longitudinal Immune Trajectory in which each immunological state is interpreted relative to those preceding it across advanced HF, LVAD support, transplantation, rejection, and CAV (Figure 5). Serial peripheral assessment would provide the longitudinal backbone, while selected high-information transitions could undergo deeper cellular, clonal, molecular, and spatial interrogation. This framework integrates immune-cell state, clonality, humoral responses, donor specificity, and tissue localization without requiring identical analytical depth at every clinical stage.

The Longitudinal Immune Trajectory remains a conceptual rather than validated surveillance framework; optimal sampling intervals, assay combinations, thresholds, and predictive performance require prospective definition. If validated, however, this approach could shift surveillance from episodic detection toward patient-specific reconstruction of how immune states emerge, persist, and evolve across time and tissue.

## 8. Conclusions

Advanced HF, durable LVAD support, and HTx are best viewed as successive phases of a continuously remodeled immune trajectory rather than immunologically isolated states. At transplantation, donor alloantigen encounters a recipient immune repertoire shaped by preceding myocardial injury, inflammation, infection, transfusion, sensitization, therapeutic interventions, and, when applicable, prolonged blood–device interaction. Subsequent graft biology reflects dynamic interactions among effector, regulatory, memory, exhaustion-associated, humoral, innate, endothelial, and stromal programs.

Neither circulating biomarkers nor histological assessment alone can fully reconstruct this complexity. Longitudinal integration of graft-injury markers, alloantibodies, cellular phenotypes, clonality, molecular programs, and tissue-resolved information may ultimately provide a more biologically coherent representation of recipient–graft immunity. The recipient immune biography and Longitudinal Immune Trajectory should therefore be regarded as conceptual frameworks whose clinical value requires prospective validation.

## Figures and Tables

**Table 1:** Immune Remodeling Across Advanced Heart Failure, LVAD Support, and Heart Transplantation: From Recipient Immune Biography to Donor-Specific Alloimmunity.

Clinical Stage	Dominant Biological Exposure	Innate Immune Changes	Adaptive Immune Changes	Memory/Remodeling Consequence	Key Unresolved Question	References
Advanced heart failure	Recurrent myocardial injury; tissue hypoperfusion and hypoxia; venous and intestinal congestion; metabolic and neurohormonal stress; recurrent decompensation	DAMP release (HMGB1, ATP, mtDNA); TLR signaling; NLRP3 inflammasome activation; IL-1β/IL-18 signaling; monocyte/macrophage activation; altered APC phenotype	Sustained inflammatory signaling; altered Th1/Th17–Treg balance; repeated lymphocyte activation; accumulation of antigen-experienced populations	Establishment of the recipient immune biography before advanced surgical therapy	Which components of HF-associated immune priming remain reversible after restoration of perfusion and mechanical unloading?	[24,25]
Early LVAD support	Surgical injury plus rapid hemodynamic improvement; initiation of continuous blood–artificial surface interaction; altered shear; hemolysis	Simultaneous removal of HF-related inflammatory stimuli and introduction of biomaterial-, complement-, platelet-, leukocyte-, and hemolysis-related signals	Early changes in lymphocyte activation and apoptotic signaling; potentially competing recovery and device-associated immune effects	Transition from HF-associated immune priming toward a new device-conditioned immune state	Which early immune changes reflect surgery and transient adaptation, and which represent the beginning of durable LVAD-associated remodeling?	[26]
Stable LVAD support—historical pulsatile/early continuous-flow devices	Chronic blood–device interaction; non-physiological flow; sustained mechanical and biomaterial exposure	Persistent monocyte activation and altered inflammatory signaling reported in historical cohorts	T-cell activation together with CD95/Fas-associated activation-induced cell death; impaired proliferative responses; altered cytokine production; reported expansion of regulatory populations	Coexistence of immune activation and impaired cellular competence; selective reshaping of lymphocyte populations	To what extent was historical “LVAD immune dysfunction” intrinsic to mechanical support versus specific to earlier device technologies?	[27]
Stable LVAD support—contemporary HeartMate 3	Long-term unloading with a fully magnetically levitated centrifugal-flow pump; reduced hemolytic and adverse hemocompatibility burden relative to earlier technology	Reduction in some HF-associated inflammatory signals may coexist with persistent blood–device interaction; longitudinal observations indicate reductions in CRP and several inflammatory cytokines	Contemporary effects on T-cell differentiation, regulation, exhaustion, memory, and clonal architecture remain incompletely characterized	Potential partial normalization of the HF immune phenotype alongside continued device-conditioned remodeling	Does contemporary HM3 support produce durable transcriptional, epigenetic, or clonal immune reprogramming, or predominantly reversible adaptation?	[6,28]
LVAD support complicated by infection	Persistent or recurrent microbial antigen exposure, particularly device-associated infection	Sustained innate-cell stimulation and inflammatory signaling; interaction between infection and device-related inflammation	Repeated antigenic stimulation may modify T-cell differentiation and memory; impaired cellular immunity may reciprocally increase infection susceptibility	Infection may become an additional component of the recipient immune biography and potentially persist immunologically beyond device explantation	Does infection during LVAD support alter the immune substrate subsequently presented to the donor graft?	[29]
Pre-transplant state after LVAD support	Resolution of some HF stimuli combined with cumulative exposure to device, infection, transfusion, sensitization, and other interventions	Innate phenotype reflects the net effect of prior HF, unloading, device exposure, and intervening inflammatory events	Variable balance among activated, regulatory, dysfunctional/exhausted-like, and memory populations; pre-existing antigen-experienced repertoire	LVAD acts as an immunological interphase in which the recipient immune biography may be partially resolved, selectively preserved, or newly remodeled	Which LVAD-associated immune states persist after transplantation and influence subsequent alloimmunity?	[30,31]
Early heart transplantation	Persistent donor HLA exposure plus donor-management, preservation, ischemia–reperfusion, and surgical injury	Tissue injury and innate activation; donor- and recipient-derived APC activity; inflammatory environment supporting alloantigen presentation	Direct, indirect, and semidirect allorecognition; costimulation; rapid expansion and differentiation of alloreactive T-cell populations; activation of T–B-cell cooperation	Transition from generalized immune priming to donor-specific alloreactivity; selection and expansion of donor-reactive clones	How does the recipient’s pre-existing immune state alter activation thresholds, clonal selection, and the early response to donor alloantigen?	[32,33]
Stable/late post-transplant state	Persistent donor antigen under chronic immunosuppression; intermittent infection, injury, rejection, and other immune challenges	Continued interaction among myeloid, NK, endothelial, and tissue-resident populations	Coexistence of effector, regulatory, inhibitory/exhaustion-associated, memory, Tfh–B-cell, and humoral programs	Progressive establishment of donor-specific cellular and humoral memory; tissue residency and clonal persistence may dissociate graft immunity from peripheral blood	What determines whether persistent alloantigen exposure evolves toward controlled adaptation, recurrent rejection, durable tissue-resident memory, or chronic graft remodeling/CAV?	[29,34,35]

Abbreviations: APC, antigen-presenting cell; ATP, adenosine triphosphate; CAV, cardiac allograft vasculopathy; CRP, C-reactive protein; DAMP, damage-associated molecular pattern; HF, heart failure; HM3, HeartMate 3; HMGB1, high-mobility group box 1; HLA, human leukocyte antigen; IL, interleukin; LVAD, left ventricular assist device; mtDNA, mitochondrial DNA; NLRP3, NLR family pyrin domain containing 3; Tfh, T follicular helper cell; TLR, Toll-like receptor; Treg, regulatory T cell.

**Table 2:** Cellular States of T-Cell Alloimmunity After Heart Transplantation: Effector Function, Regulation, Exhaustion, and Memory.

Cellular State	Representative Phenotype/Molecular Features	Principal Drivers	Dominant Functional Role	Relevance Within the Graft	Critical Interpretative Caveat	References
Th1 effector cells	CD4+; T-bet-associated program; IFN-γ production	Donor-antigen recognition; TCR signaling; costimulation; inflammatory cytokine environment	Macrophage activation; amplification of antigen presentation and inflammatory signaling	Promotes endothelial and parenchymal inflammation and reinforces local APC activation	Presence or abundance alone does not establish the magnitude of tissue injury; function depends on activation state and localization	[65,70]
Th17 effector cells	CD4+; RORγt-associated program; IL-17-associated signaling	Inflammatory cytokine milieu accompanying alloantigen recognition	Leukocyte recruitment and maintenance of tissue inflammation	May sustain inflammatory cellular networks within the graft	Th17-associated phenotype is not necessarily stable; functional programs may overlap with other activated CD4+ states	[71,72]
Cytotoxic CD8+ T cells	CD8+; perforin; granzymes; FasL; IFN-γ; TNF	Donor-antigen recognition and productive T-cell activation	Direct cytotoxicity and inflammatory cytokine production	Injury to graft parenchymal and vascular cells; participation in cellular rejection	Cytotoxic potential cannot be inferred from CD8+ cell number alone; activation state, clonality, and tissue position are critical	[65,73]
Regulatory T cells (Tregs)	CD4+CD25^high^FOXP3+; CTLA-4; CD39–CD73; IL-10/TGF-β-associated mechanisms	Regulatory differentiation; antigen exposure; local cytokine and metabolic conditions	Suppression of conventional T-cell activation; reduction in APC costimulatory capacity; metabolic and cytokine-mediated restraint	May limit pathogenic alloimmunity at sites of donor-antigen recognition	FOXP3+ cell abundance does not establish effective regulation; suppressive competence, stability, localization, and Treg/effector balance are more informative	[70,74]
Activated inhibitory-receptor-positive T cells	PD-1+, with possible TIM-3, LAG-3, or TIGIT expression	Recent or sustained TCR engagement and activation	May represent an activated state accompanied by engagement of inhibitory pathways	Potentially reflects ongoing antigen encounter within blood or graft	PD-1 expression alone should not be interpreted as T-cell exhaustion; PD-1 is rapidly induced after activation	[75]
Exhaustion-associated/dysfunctional T cells	Co-expression of inhibitory receptors; altered transcriptional and metabolic programs; reduced proliferative and cytokine capacity	Persistent or repetitive alloantigen stimulation; chronic TCR signaling; modified by immunosuppressive therapy	Attenuation of effector function despite continued antigen recognition	May restrain chronic alloimmune injury while preserving residual donor-reactive potential	Surface markers alone are insufficient; functional competence, transcriptional state, clonality, and tissue localization are required to distinguish exhaustion from activation or adaptive restraint	[76,77]
Central-memory T cells (T_CM)	Antigen-experienced memory phenotype with lymphoid-homing capacity and substantial proliferative reserve	Previous antigen exposure followed by memory differentiation	Long-term maintenance of antigen-experienced clones and capacity for secondary expansion	Provides a reservoir capable of renewed donor-reactive responses	Circulating T_CM do not capture the complete memory repertoire maintained within peripheral tissues or the graft	[78,79]
Effector-memory T cells (T_EM)	Antigen-experienced phenotype positioned for rapid peripheral effector responses	Previous activation and differentiation toward peripheral memory	Rapid recall response and effector function following antigen re-encounter	May contribute rapidly to graft-directed inflammation following renewed stimulation	Phenotypically defined T_EM populations may contain heterogeneous transcriptional and functional states	[53,80]
Tissue-resident memory T cells (T_RM)	Tissue-retention/residency program; maintained within non-lymphoid tissue without continuous recirculation	Tissue entry followed by local retention and adaptation to the graft microenvironment	Rapid local recall responses and persistent immune surveillance	Positioned near donor antigen, APCs, endothelial cells, and stromal populations; may sustain locally organized graft immunity	Peripheral blood may substantially underestimate this compartment; tissue sampling and spatial localization are required	[44,81]
Heterologously cross-reactive memory T cells	Pre-existing antigen-experienced clones capable of cross-reactivity with allogeneic HLA	Previous infections or environmental antigen exposure before transplantation	Rapid alloimmune responsiveness with reduced dependence on naïve-cell activation requirements	Provides a mechanistic link between the pre-transplant immune biography and donor responsiveness	Previous immune memory is not necessarily donor-specific; cross-reactivity must not be inferred solely from the presence of memory cells	[82,83]
Donor-reactive clonal memory	Expanded donor-responsive clonotypes persisting across effector and/or memory states	Persistent donor alloantigen and post-transplant clonal selection	Long-term preservation of donor-specific immune potential	May persist in circulation and/or become compartmentalized within the graft	Phenotype alone cannot establish lineage; longitudinal TCR clonotyping is required to determine whether different states derive from the same alloreactive clone	[84,85]

Abbreviations: APC, antigen-presenting cell; HLA, human leukocyte antigen; IL, interleukin; Treg, regulatory T cell.

**Table 3:** The Tfh–B-Cell–Plasma-Cell–DSA Axis: Cellular Architecture and Effector Mechanisms of Humoral Alloimmune Memory.

Component/Stage	Principal Biological Role	Key Molecular Interactions/Mechanisms	Contribution to Alloimmune Memory or Graft Injury	Potential Measurable Readout	Critical Interpretative Limitation	References
T follicular helper (Tfh) cells	Translate T-cell recognition of processed donor antigen into B-cell selection, proliferation, and maturation	Cognate T–B interaction; CD40L–CD40; IL-21; follicular migration and sustained B-cell help	Determines which donor-reactive B-cell clones receive survival and differentiation signals and subsequently enter memory or antibody-secreting compartments	Circulating Tfh-like populations; T-cell phenotype/transcriptome; TCR clonality	Circulating Tfh-like cells may not reproduce the phenotype or activity of Tfh cells participating in lymphoid or tissue-localized responses	[114,115]
Activated donor-reactive B cells	Recognize antigen, receive T-cell help, proliferate, and enter differentiation pathways; may also function as APCs	BCR-mediated antigen recognition; antigen processing/presentation; CD40–CD40L; IL-21-dependent Tfh help	Connects donor-antigen recognition with germinal-center evolution and humoral memory formation	Activated B-cell phenotype; BCR repertoire; single-cell transcriptomics	B-cell abundance alone does not establish donor specificity, clonal evolution, or functional state	[41,116]
Germinal-center B cells	Undergo clonal expansion and selection, progressively refining the donor-reactive B-cell repertoire	Somatic hypermutation; affinity-dependent selection; class-switch recombination; continued Tfh-mediated selection	Generates increasingly selected clones capable of entering memory B-cell or plasma-cell compartments	BCR sequencing; somatic-hypermutation patterns; clonal lineage reconstruction	The germinal-center reaction is poorly captured by routine peripheral blood surveillance	[72,117]
Memory B cells	Provide a persistent reservoir of antigen-experienced clones capable of rapid re-expansion and differentiation after renewed stimulation	Long-term maintenance of selected B-cell clonotypes; rapid recall after antigen re-encounter	Encodes durable donor-reactive humoral memory even in the absence of substantial ongoing antibody production	Memory B-cell phenotyping; donor-reactive assays; BCR clonotyping	Low or absent circulating DSA does not exclude an underlying donor-reactive memory B-cell reservoir	[118]
Plasmablasts	Provide an actively differentiating, antibody-secreting B-cell compartment	Terminal B-cell differentiation and active immunoglobulin secretion	May reflect active or evolving humoral immune responses	Circulating plasmablast frequency; single-cell transcriptomics; BCR sequencing	A transient circulating plasmablast response should not be equated with established long-lived humoral memory	[119,120]
Long-lived plasma cells	Maintain sustained antibody secretion with relatively limited dependence on the signals required for initial B-cell activation	Persistent terminally differentiated antibody secretion	Can sustain DSA production independently of the size of the circulating B-cell compartment	Circulating DSA as an indirect output; plasma-cell characterization where accessible	Peripheral B-cell depletion does not necessarily eliminate established plasma-cell populations; DSA does not directly identify its cellular source	[120]
Donor-specific antibodies (DSA)	Recognize donor HLA and connect donor-specific adaptive memory with graft-directed effector mechanisms	Binding to donor HLA; capacity to engage complement and Fc receptor-bearing cells	Provides donor specificity to downstream complement-, NK-cell-, and endothelial-mediated injury	Solid-phase HLA antibody assays; DSA specificity and persistence; MFI	MFI is not a direct measure of antibody concentration or pathogenicity; biological effects depend on specificity, persistence, subclass, avidity, complement engagement, antigen density, and accessibility	[121,122]
Complement	Amplifies antibody-triggered inflammatory and vascular responses	Classical complement pathway; inflammatory complement fragments; terminal C5b–9complex	Promotes endothelial activation, vascular permeability, leukocyte recruitment, and microvascular injury	Complement-binding antibody assays; tissue complement deposition where assessed	Complement activation represents only one potential effector pathway of DSA; absence of a single complement readout does not reconstruct the complete humoral response	[123]
NK cells	Convert graft-bound antibody recognition into cellular effector activity	FcγRIIIa/CD16engagement by Fc portions of graft-bound IgG; cytotoxic and inflammatory signaling	Mediates ADCC and links adaptive humoral recognition with innate cellular graft injury; interacts with endothelial and myeloid compartments	NK-cell phenotype/activation state; tissue localization; potentially single-cell profiling	NK-cell number in blood does not establish antibody-dependent activity within the graft; proximity to antibody-coated vascular targets is biologically important	[124]
Graft endothelium	Principal vascular interface between circulating DSA and donor tissue; active regulator of subsequent inflammation	Donor HLA ligation; complement exposure; cytokine signaling; interaction with NK cells and monocytes; altered adhesion molecules and chemokines	Converts antibody recognition into endothelial activation, leukocyte recruitment, microvascular inflammation, and potentially chronic vascular remodeling	Histology/immunopathology; endothelial transcriptomic signatures; tissue molecular and spatial profiling	Endothelium should not be interpreted as a passive antibody target; apparently similar circulating DSA profiles may produce different tissue responses	[125]
Integrated humoral alloimmune network	Couples donor-antigen recognition, cellular selection, memory formation, antibody production, and tissue effector mechanisms	Tfh → B cell → germinal center → memory B cell/plasma cell → DSA → complement/NK cell → endothelium	Provides a mechanistic continuum from adaptive immune memory to antibody-mediated and chronic vascular graft injury	Integrated TCR/BCR sequencing, DSA profiling, single-cell and spatial tissue analysis	No single circulating biomarker reconstructs the entire pathway; similar DSA profiles may arise from biologically distinct upstream cellular architectures	[41]

Abbreviations: ADCC, antibody-dependent cellular cytotoxicity; APC, antigen-presenting cell; BCR, B-cell receptor; CD40L, CD40 ligand; DSA, donor-specific antibody; FcγRIIIa, Fc gamma receptor IIIa; HLA, human leukocyte antigen; IgG, immunoglobulin G; IL-21, interleukin-21; MFI, mean fluorescence intensity; NK, natural killer; TCR, T-cell receptor; Tfh, T follicular helper.

**Table 4:** Cellular Architecture of the Transplanted Heart: Immune, Vascular, and Stromal Components of the Graft Ecosystem.

Cellular Component	Origin/Compartment	Principal Biological Functions	Key Cellular Interactions	Contribution to Rejection and Chronic Remodeling	Why Peripheral Blood is Insufficient	References
Tissue-resident memory T cells (T_RM)	Recipient-derived; retained within non-lymphoid graft tissue	Local immune surveillance; rapid recall responses; persistent recognition of antigen within the graft	APCs; endothelial cells; macrophages; fibroblasts; other stromal populations	May sustain locally organized inflammatory and donor-reactive responses without requiring repeated recruitment from the circulation	T_RM are defined partly by tissue residency and may be poorly represented or absent in peripheral blood; circulating memory populations therefore incompletely reflect graft memory	[53]
Graft-infiltrating memory CD4+ and CD8+ T cells	Recipient-derived; recruited to and/or retained within graft tissue	Cytokine production, cytotoxicity, inflammatory amplification, memory responses	Endothelium; APCs; macrophages; fibroblasts	Human CAV tissue contains memory T cells with inflammatory, profibrotic, activation, and tissue-residency features, potentially linking persistent adaptive immunity with chronic remodeling	Circulating counterparts may not share the same activation, transcriptional, or tissue-residency programs	[149]
Tissue B cells/donor-reactive B-cell clones	Recipient-derived; compartmentalized within graft immune niches	Antigen presentation; maintenance or reactivation of humoral memory; potential local T–B-cell interaction	T cells/Tfh-related populations; APCs; stromal cells; potentially plasma-cell compartments	May provide a tissue reservoir of donor-reactive humoral immunity and contribute to persistent local immune organization	Blood B-cell repertoire and serum DSA may not reproduce graft B-cell clonality; B-cell compartmentalization may be particularly marked in CAV	[150]
Donor-derived cardiac macrophages	Donor origin; resident cardiac macrophage compartment persisting after transplantation	Tissue surveillance; injury sensing; antigen handling; cytokine/chemokine production; debris clearance; repair	Recipient leukocytes; APCs; endothelial cells; fibroblasts	Contribute to the initially chimeric innate immune landscape and may shape subsequent recipient immune recruitment and tissue responses	Cannot be reconstructed from circulating monocytes; tissue analysis is required to establish origin, state, and localization	
CCR2+ donor macrophages	Donor-derived cardiac macrophage subset	Pro-inflammatory signaling; recruitment and activation of recipient immune populations	Recipient APCs and infiltrating myeloid cells; neighboring tissue cells	Can promote rejection through MYD88-dependent inflammatory signaling and recruitment of recipient APCs	Their biological relevance depends on tissue origin and local state, neither of which is identifiable from routine peripheral monocyte measurements	
CCR2− donor macrophages	Donor-derived resident macrophage compartment	Tissue-homeostatic and repair-associated functions within the resident macrophage network	Cardiomyocytes; endothelial and stromal populations; other immune cells	Functionally distinct from CCR2+ macrophages; may contribute differently to tissue homeostasis and responses to injury	Peripheral blood cannot determine persistence or functional state of donor-resident macrophage populations	
Recipient monocytes/monocyte-derived macrophages	Recipient-derived; recruited from circulation into the graft	Injury sensing; antigen processing/presentation; inflammatory cytokine and chemokine production; phagocytosis; repair/fibrotic signaling	T cells; donor macrophages; endothelium; fibroblasts; complement pathways	Participate in acute rejection and chronic remodeling; may acquire distinct interferon-, inflammasome-, or tissue-conditioned states	Circulating monocyte phenotype does not predict the transcriptional and functional state acquired after entry into the graft	[151]
Natural killer (NK) cells	Predominantly recipient-derived; circulating and graft-infiltrating	Fc-dependent cytotoxicity; IFN-γ production; amplification of innate–adaptive communication	DSA-coated endothelium via FcγRIIIa/CD16; monocytes/macrophages; endothelial cells	Convert antibody recognition into cellular injury through ADCC and participate in microvascular inflammatory networks	Peripheral NK-cell abundance does not establish activation or proximity to antibody-coated vascular targets within the graft	[121,124]
Endothelial cells	Donor-derived vascular compartment	Alloantigen presentation/interface; control of leukocyte adhesion and trafficking; chemokine production; response to inflammatory and antibody-mediated signals	T cells; NK cells; monocytes/macrophages; DSA/complement; VSMCs	Central target and active amplifier of cellular and humoral alloimmunity; persistent activation may link recurrent immune injury with microvascular dysfunction and CAV	Circulating biomarkers provide indirect evidence but cannot reproduce endothelial state, HLA expression, leukocyte interactions, or vascular localization	[152]
Fibroblasts	Donor-derived stromal compartment, with phenotype shaped by local injury and inflammation	Extracellular-matrix production; cytokine, chemokine, and growth-factor signaling; organization of tissue remodeling	Macrophages; T cells; endothelial cells; other stromal populations	Connect immune injury with fibrosis; may influence immune-cell localization, persistence, and function while responding to inflammatory signals	Their activation state and spatial relationship with immune cells are fundamentally tissue-specific	[153]
Vascular smooth muscle cells (VSMCs)	Donor-derived coronary vascular wall	Maintenance of vascular structure; phenotypic adaptation to inflammatory and growth signals; proliferative and matrix-remodeling responses	Endothelial cells; macrophages; lymphocytes; fibroblast/stromal signals	Phenotypic transition and proliferation contribute to neointimal expansion and the structural evolution of CAV	Peripheral immune measurements cannot capture VSMC phenotypic transition or its relationship to local vascular inflammation	[123]
Immune–stromal cellular neighborhood	Spatially organized multicellular graft niche	Integrates antigen recognition, inflammatory signaling, cytotoxicity, immune regulation, repair, fibrosis, and vascular remodeling	Reciprocal interactions among T/B/NK cells, macrophages, endothelium, fibroblasts, and VSMCs	May sustain or redirect graft inflammation and progressively convert previous immune injury into persistent vascular and fibrotic remodeling	Cannot be reconstructed from cell counts or blood biomarkers; requires tissue-resolved single-cell and particularly spatial approaches	[122]

Abbreviations: ADCC, antibody-dependent cellular cytotoxicity; APC, antigen-presenting cell; CAV, cardiac allograft vasculopathy; CCR2, C-C chemokine receptor type 2; DSA, donor-specific antibody; FcγRIIIa, Fc gamma receptor IIIa; IFN-γ, interferon gamma; MYD88, myeloid differentiation primary response 88; NK, natural killer; Tfh, T follicular helper; T_RM, tissue-resident memory T cell; VSMC, vascular smooth muscle cell.

**Table 5:** From Clinical Surveillance to Spatial Immunology: Translational Readiness and a Multilevel Framework for Precision Immune Phenotyping in Heart Transplantation.

Level of Assessment	Representative Methods/Measurements	Biological Information Obtained	Principal Strength	Critical Limitation	Role in the Longitudinal Immune Trajectory	References
Non-HLA antibodies and autoantibodies	Antibody assays targeting non-HLA antigens and selected autoantigens; antigen-specific immunoassays or multiplex platforms	Humoral reactivity beyond conventional HLA-directed DSA, including potential endothelial, vascular, and tissue-directed autoreactivity	May identify a complementary humoral component of graft injury not captured by conventional HLA-DSA assessment	Assay standardization is limited; antigen panels, clinically relevant thresholds, pathogenicity, and incremental prognostic value remain incompletely established	May complement longitudinal DSA profiling, particularly in otherwise unexplained graft dysfunction or suspected antibody-mediated injury without detectable HLA-DSA	[163]
Circulating cytokines and chemokines	Multiplex cytokine/chemokine assays; ELISA; high-sensitivity immunoassays; targeted proteomic panels	Systemic inflammatory and immune-activation state, including Th1-, myeloid-, endothelial-, and chemotactic signaling programs	Dynamic and mechanistically informative; permits simultaneous assessment of multiple interacting inflammatory pathways	Limited graft and rejection specificity; strongly influenced by infection, systemic inflammation, renal dysfunction, tissue injury, and immunosuppressive therapy	Serial trajectories may identify transitions between relative immune quiescence and inflammatory activation and complement graft-specific injury biomarkers	[164]
Soluble ST2 (sST2)	Serum/plasma sST2 measurement using standardized immunoassays	Myocardial stress and inflammatory–fibrotic signaling associated with tissue injury and adverse remodeling	Readily measurable circulating biomarker with potential prognostic value and suitability for serial assessment	Not specific for alloimmune activation or rejection; concentrations may reflect myocardial stress and systemic inflammatory processes unrelated to graft-directed immunity	May provide a complementary longitudinal measure of graft stress and inflammatory–fibrotic burden when interpreted alongside DSA, dd-cfDNA, imaging, and clinical findings	[165]
Extracellular vesicles and exosomes	EV isolation and characterization; extracellular-vesicle proteomics; miRNA/RNA profiling; flow cytometry; nanoparticle-based or other emerging vesicle assays	Cell-to-cell molecular communication and potentially cell- or tissue-of-origin information through protein, lipid, mRNA, miRNA, and other molecular cargo	Potential to capture biologically rich, tissue- and cell-associated molecular signals not represented by conventional soluble biomarkers	Major pre-analytical and analytical heterogeneity; isolation and normalization remain incompletely standardized; clinically validated thresholds are lacking	Research-level longitudinal profiling may help characterize endothelial, immune, and graft-derived molecular transitions preceding or accompanying overt rejection or remodeling	[166]
Circulating injury and humoral biomarkers	dd-cfDNA; DSA; circulating inflammatory mediators	Graft-cell injury; donor-directed humoral recognition; systemic inflammatory activity	Minimally invasive and suitable for frequent longitudinal sampling	Injury does not identify the responsible immune population; DSA do not reveal the cellular architecture maintaining antibody production; absence of an abnormal signal does not establish immune quiescence	Provides repeatable surveillance points and identifies transitions requiring deeper cellular or tissue-level investigation	[167]
Conventional and high-dimensional immune phenotyping	Multiparameter/high-dimensional flow cytometry; enumeration of naïve, activated, effector, regulatory, inhibitory-receptor-positive, memory, B-cell, NK-cell, and monocyte populations	Composition and phenotypic state of circulating immune compartments	Repeatable; cell-specific; permits longitudinal assessment of changes in major immune populations	Phenotype does not necessarily establish function, clonality, donor specificity, or tissue localization; PD-1 and other inhibitory receptors are context dependent	Defines temporal changes in circulating immune-cell composition from advanced HF through LVAD support and post-transplant follow-up	[168,169]
Peripheral gene-expression profiling	Whole-blood or PBMC transcriptional signatures	Systemic transcriptional programs associated with immune activation and graft-related biological states	Captures coordinated molecular signals not apparent from cell counts alone and can complement other non-invasive markers	Bulk signals average across heterogeneous cell populations and may not identify the cellular source or reproduce graft biology	Provides a molecular layer complementary to cellular phenotyping and circulating injury markers	[139]
Single-cell transcriptomics	scRNA-seq of PBMCs and/or graft-derived cells	Cell-specific transcriptional states; activation, interferon-response, cytotoxic, regulatory, metabolic, and exhaustion-associated programs	Resolves heterogeneity hidden within conventional phenotypic populations and bulk measurements	Provides a molecular snapshot; transcriptional state alone does not establish cellular lineage, previous state, donor specificity, or anatomical context	Identifies stage-specific immune states and determines how cellular programs change across HF, LVAD support, transplantation, rejection, and chronic graft disease	[151]
TCR clonotyping	TCR sequencing integrated with immune phenotyping or single-cell transcriptomics	Clonal expansion, persistence, and relationships among T-cell states across time and compartments	Adds cellular history and allows potentially related T-cell states to be linked longitudinally	Clonality alone does not establish functional state or necessarily prove donor specificity; interpretation requires integration with phenotype and clinical context	Potentially tracks persistent or expanding T-cell clonotypes from pre-transplant states through alloantigen exposure, effector differentiation, inhibitory programs, and memory	[170]
BCR clonotyping	BCR sequencing; paired single-cell transcriptome–BCR analysis	B-cell clonal expansion, somatic evolution, and relationships among activated, memory, plasmablast, and antibody-secreting populations	Reconstructs the cellular lineage underlying humoral memory more directly than circulating DSA alone	Circulating repertoire may incompletely represent lymphoid or graft-resident B-cell compartments; clonal detection does not itself establish pathogenicity	Links donor-reactive humoral evolution to memory formation, DSA emergence, persistence, and potential tissue compartmentalization	[171]
Graft molecular profiling	Endomyocardial-biopsy transcriptomics; molecular biopsy assessment	Tissue-level immune, endothelial, stromal, and injury-associated molecular programs	Directly interrogates the organ in which alloimmune injury occurs and can reveal biology not represented in peripheral blood	Invasive; spatially limited sampling; bulk tissue analysis may still obscure cellular heterogeneity and anatomical relationships	Anchors circulating immune trajectories to the biological state of the graft at clinically informative time points	[172]
Graft single-cell profiling	scRNA-seq of graft-derived cells, ideally integrated with TCR/BCR sequencing	Cell-specific immune and stromal states within the disease site; macrophage heterogeneity; tissue T- and B-cell states; endothelial and fibroblast programs	Resolves the cellular composition and functional heterogeneity of the graft ecosystem	Tissue dissociation removes anatomical relationships; biopsy samples represent only a small portion of a spatially heterogeneous organ	Determines whether circulating cellular states are reproduced, transformed, enriched, or absent within the graft	[55]
Spatial transcriptomics and spatial proteomics	Spatially resolved RNA and protein profiling of myocardial or vascular tissue	Anatomical localization of cellular states; immune–immune and immune–stromal neighborhoods; spatially restricted signaling relationships	Restores tissue architecture and allows cellular state to be interpreted in relation to neighboring cells and anatomical structures	Limited tissue availability; sampling constraints; methodological complexity; currently unsuitable for frequent routine longitudinal surveillance	Defines where persistent immune states reside and how immune, endothelial, and stromal populations organize into rejection- or remodeling-associated niches	[173]
Integrated multimodal immune phenotype	Combined blood biomarkers, immune phenotyping, transcriptomics, TCR/BCR clonotyping, tissue molecular profiling, and spatial analysis	Multiscale reconstruction of immune state linking injury, phenotype, cell state, clonality, tissue localization, and cellular interaction	Moves beyond isolated biomarkers toward mechanistic interpretation of the recipient’s evolving immune state	Requires harmonized sampling, temporal alignment, analytical integration, prospective validation, and clinically feasible prioritization of assays	Provides the analytical basis for patient-specific longitudinal immune reconstruction rather than classification from isolated measurements	[174]
Proposed Longitudinal Immune Trajectory	Serial multimodal assessment across advanced HF → pre-LVAD → stable LVAD → pre-HTx → early post-HTx → stable late post-HTx → rejection and/or CAV	Patient-specific reconstruction of how immune states emerge, resolve, persist, or transform across successive clinical stages	Integrates timewith cellular state, lineage, donor-directed immunity, and tissue context; treats each stage relative to the preceding biological state	Conceptual framework requiring prospective validation; optimal sampling intervals, assay combinations, thresholds, and predictive value remain to be established	Proposed framework for moving from event-centered surveillance toward longitudinal precision immune phenotyping	[36,175]

Footnote: Translational status reflects the current general level of clinical implementation rather than a formal guideline-based recommendation. Availability, analytical standardization, and clinical use of individual assays may vary among transplant centers and healthcare systems. Research and investigational approaches are presented for their potential contribution to longitudinal immune phenotyping and should not be interpreted as validated replacements for current guideline-based post-transplant surveillance. The biomarkers included in this framework interrogate biologically distinct and complementary dimensions of recipient–graft biology and should not be considered interchangeable: dd-cfDNA predominantly reflects graft-cell injury; DSA and non-HLA antibodies characterize distinct components of humoral immune reactivity; circulating cytokines and chemokines provide information on systemic inflammatory and immune activation; sST2 reflects myocardial stress and inflammatory–fibrotic signaling; EVs may convey cell- and tissue-associated molecular information; and TTV load has been investigated as an indirect marker of the net state of immunosuppression and immune competence. None of these measurements, when considered in isolation, reconstructs the complete immunological state of the recipient or graft. Their integration within the proposed Longitudinal Immune Trajectory is therefore intended primarily as a translational research framework for multimodal and serial immune phenotyping rather than as a treatment algorithm or replacement for established clinical, histological, immunological, and molecular surveillance. Prospective studies are required to determine whether such integrated trajectories improve prediction of rejection, graft remodeling, infection, or other clinically relevant outcomes and whether they can safely support individualized immunosuppressive management. Abbreviations: BCR, B-cell receptor; CAV, cardiac allograft vasculopathy; dd-cfDNA, donor-derived cell-free DNA; DSA, donor-specific antibody; ELISA, enzyme-linked immunosorbent assay; EV, extracellular vesicle; HF, heart failure; HLA, human leukocyte antigen; HTx, heart transplantation; LVAD, left ventricular assist device; miRNA, microRNA; mRNA, messenger RNA; NK, natural killer; PBMC, peripheral blood mononuclear cell; scRNA-seq, single-cell RNA sequencing; sST2, soluble suppression of tumorigenicity 2; TCR, T-cell receptor; TTV, Torque Teno Virus.

## Data Availability

No new data were created or analyzed in this study. Data sharing is not applicable to this article.

## Abbreviations

The following abbreviations are used in this manuscript:

ADCC	antibody-dependent cellular cytotoxicity
APC	antigen-presenting cell
ATP	adenosine triphosphate
BCR	B-cell receptor
C3	complement component 3
C9	complement component 9
CAV	cardiac allograft vasculopathy
CCR2	C-C chemokine receptor type 2
CD	cluster of differentiation
CD40L	CD40 ligand
CRP	C-reactive protein
CTLA-4	cytotoxic T-lymphocyte-associated protein 4
DAMP	damage-associated molecular pattern
dd-cfDNA	donor-derived cell-free DNA
DSA	donor-specific antibody
FcγRIIIa	Fc gamma receptor IIIa
FOXP3	forkhead box P3
HF	heart failure
HM3	HeartMate 3
HMGB1	high-mobility group box 1
HLA	human leukocyte antigen
HTx	heart transplantation
IFN-γ	interferon gamma
IgG	immunoglobulin G
IL	interleukin
LAG-3	lymphocyte-activation gene 3
LVAD	left ventricular assist device
MFI	mean fluorescence intensity
MHC	major histocompatibility complex
mtDNA	mitochondrial DNA
mTOR	mechanistic target of rapamycin
MYD88	myeloid differentiation primary response 88
NFAT	nuclear factor of activated T cells
NK	natural killer
NLRP3	NLR family pyrin domain containing 3
PBMC	peripheral blood mononuclear cell
PD-1	programmed cell death protein 1
PD-L1	programmed death-ligand 1
RNA-seq	RNA sequencing
scRNA-seq	single-cell RNA sequencing
TCR	T-cell receptor
T_CM	central memory T cell
T_EM	effector memory T cell
Tfh	T follicular helper cell
TGF-β	transforming growth factor beta
Th1	T helper 1 cell
Th17	T helper 17 cell
TIGIT	T-cell immunoreceptor with immunoglobulin and ITIM domains
TIM-3	T-cell immunoglobulin and mucin-domain containing-3
TLR	Toll-like receptor
TNF	tumor necrosis factor
Treg	regulatory T cell
T_RM	tissue-resident memory T cell
UMAP	Uniform Manifold Approximation and Projection
VSMC	vascular smooth muscle cell
